#  Identification and Simultaneous Determination of Twelve Active Components in the Methanol Extract of Traditional Medicine Weichang’an Pill by HPLC-DAD-ESI-MS/MS 

**Published:** 2013

**Authors:** Jingze Zhang, Wenyuan Gao, Zhen Liu, Zhidan Zhang

**Affiliations:** *School of Pharmaceutical Science and Technology, Tianjin University, Tianjin 300072, China. *

**Keywords:** HPLC-DAD-ESI-MS/MS, Quantitative analysis, Traditional medicine, Weichang’an Pill.

## Abstract

Weichang’an (WCA) pill, a traditional Chinese patent medicine consisting of ten Chinese medicinal herbs, has been used to treat irritable bowel syndrome and functional dyspepsia for several decades. In this study, twelve bioactive constituents in the methanol extract of WCA were accurately identified since MS/MS fragmentation behavior of the references and the standards by using HPLC-DAD-ESI-MS/MS analysis and a reliable and accurate method for the simultaneous determination was developed. Twelve active components including costunolide and dehydrodehydrocostus lactone from the principal herb *Radix Aucklandiae*; naringin, hesperidin and neohesperidin from *Fructus Aurantii*; magnolol and honokiol from the ministerial herbs *Cortex Magnoliae officinalis*; aloe-emodin, rhein, emodin, chrysophanol and physcion from adjunctive and messenger herb *Radix et Rhizoma Rhei *were analyzed in the samples. The chromatographic separation was performed on a Kromasil C_18 _column with gradient elution of acetonitrile-methanol and 1.0% acetic acid water. In this condition, linearity, inter- and intra-day precision and accuracy were within acceptable ranges. The developed method showed satisfactory precision and accuracy with overall intra- and inter-day variations of 0.68-1.33% and 0.67-2.05% respectively, and the overall recoveries of 97.54-102.69% for twelve compounds. The proposed approach was successfully applied as a powerful tool for the quality control of WCA pill.

## Introduction

In recent years, traditional Chinese medicine (TCM) has been widely used in many countries and attracts considerable attention due to its special effectiveness and low toxicity. Commercially available TCM formula is usually composed of several herbs with numerous constituents. Thus, the analysis of such a complex mixture brings a great challenge to pharmaceutical analysts. Weichang’an (WCA) pill, a Chinese traditional patent medicine, consists of ten Chinese medicinal herbs including *Radix Aucklandiae *(the dried root of *Aucklandiae lappa *Dence.)*, Lignum Aquilariae Resinatum *(the resin lignum of *Aquilaria sinensis *(Lour.) Gilg)*, Lignum Aantali Albi *(the resin lignum of *Santalum album *L.)*, Fructus Aurantii *(the closely mature fruit of *Citrus auranfium *L.)*, Cortex Magnoliae officinalis *(the bark of *Magnolia officinalis *Rehd. et wils.), *Radix et Rhizoma Rhei *(the dried rhizome and root of *Rheum palmatum *L.), *Rhizoma chuanxiong *(the dried rhizome of *Ligusticum chuanxiong *Hort.)*, Semen Crotonis Plulveratum *(the seed powder of *Croton tiglium *L.), *Fructus Jujubae *(the dry mature fruit of *Ziziphus jujuba *Mill.) and *Moschus. *These herbs are milled into fine powder, *mixe*d and made into water pills, which has been used for the treatment of various gastrointestinal (GI) diseases such as diarrhoea, enteritis, dysentery, irritable bowel syndrome, nausea, vomiting, indigestion, abdominal pain and distension for several decades (1, 2). It possesses the properties of eliminating damp pathogen, regulating vital energy to alleviate pain, and removing food from the stomach and intestine due to the indigestion (3). Our previous research had been reported that the methanol extract of WCA is able to inhibit diarrhoea, increase normal gastrointestinal transit, and decrease gastrointestinal transit induced by neostigmine. The results suggested that the methanol extract of WCA might have a bidirectional role in the GI tract (4). Despite the popular medicinal usage of WCA, there has been no fully integrated study of the constituents in the formula. Multi-constituents analysis by liquid chromatography coupled with diode array detector and electrospray ionization tandem mass spectrometry (LC/DAD/ESI/MS/MS) is a simple and powerful analytical tool for the analysis of the known compounds in complex matrix (5-8). In present study, twelve components in the methanol extract WCA pill were identified accurately and a reliable analytical method for the simultaneous determination of the constituents was developed by HPLC-DAD. Among of which costunolide, dehydrocostus lactone were sesquiterpenoids from the principal herb *R. Aucklandiae *(9); naringin, hesperidin and neohesperidin were flavonoid glycosides from the ministerial herbs *F. Aurantii *(10); magnolol and honokiol were lignanoids from the ministerial herbs *C. Magnoliae officinalis *(11) and aloe-emodin, rhein, emodin, chrysophanol and physcion were anthraquinones from the adjunctive and messenger herb *R. et R. Rhei *(12). To the best of our knowledge, it is the first time that the main bioactive constituents has been simultaneously determined from the principal, ministerial, adjunctive and messenger herbs to evaluate the quality of the TCM production.

## Experimental

*Chemicals and reagents*

HPLC grade acetonitrile and methanol were purchased from Fisher (USA). Water was purified by a Milli-Q water purification system (Millipore, USA). Other reagents were of analytical grade.

**Figure 1:**
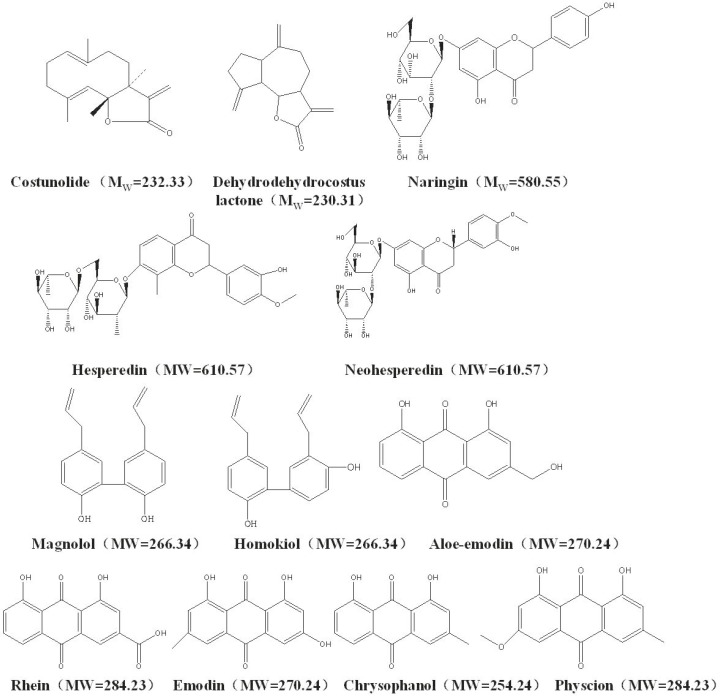
Chemical structures of the twelve bioactive compounds to be determined in WCA

Standards including costunolide, dehydrocostus lactone, naringin, hesperidin, neohesperidin, magnolol, honokiol, aloe-emodin, rhein, emodin, chrysophanol and physcion were purchased from the National Institute for the Control of Pharmaceutical and Biological Products (Beijing, China). All the twelve reference compounds have over 98% purity (see their chemical structures in Figure 1). All the voucher specimens (Voucher No. WCAW-090601–060610) were available in the herbarium of Research Center of Tianjin Zhongxin Pharmaceuticals.

*HPLC analysis*

All analyses were performed on an Agilent 1100 liquid chromatography system (Agilent Technologies, USA), equipped with a quaternary pump, an online degasser, and a column temperature controller, coupled with an DAD (Alltech Associates ,USA) as the detector. The analytical column was a Kromasil C_18_ (250 mm × 4.6 mm *i.d.*, 5 μm particle size) and the column temperature was kept at 35°C. The mobile phase was a linear gradient prepared from acetonitrile (A), methanol (B), and water (containing 1% acetic acid) (C). The composition of the gradient was A-B-C, 4.3:0.7:95 at 0 min, 20:2.5:77.5 at 15 min, 22:3.5:74.5 at 40 min, 50:8:42 at 70 min, 69:11:20 at 100 min and then the system was returned to initial conditions. The flow rate was 0.8 mL/min, and the injection volume was 20 μL.

*HPLC-ESI-MS/MS analysis*

Samples were analyzed using an Agilent HPLC–MS system containing a surveyor auto-sampling system (Agilent Technologies, USA) and an LC/MSD Trap XCT electrospray ion trap mass spectrometer. Source settings used for the ionization were as follows: nebulizer gas flow, 70.00 psi; dry gas flow, 11.00 L/min; electrospray voltage of the ion source, 3000 V; capillary temperature, 350°C; capillary exit, - 158.5 V; skimmer, 40 V. Nitrogen (> 99.99%) and He (> 99.99%) were utilized as sheath and lamping gas, respectively. The full scan of ions ranging from m/z 100 to 1000 in the positive and negative ion mode was carried out. The fragment ions were obtained using collision energy of 35% for both MS^2^ and MS^3^ experiments. Analyses were conducted at ambient temperature and the data were operated on the Xcalibur software. 

*Stock and working solutions*

Each accurately weighed standard was dissolved in methanol respectively, and various standard solutions were obtained through diluting the stock solution in a series of concentrations in order to make the calibration curves. 

A stock solution containing the twelve standards (costunolide 135.8 μg/mL, dehydrodehydrocostus lactone 143.7 μg/mL, naringin 804.0 μg/mL, hesperidin 30.8 μg/mL, neohesperidin 604.2 μg/mL, magnolol 866.4 μg/mL, honokiol 700.8 μg/mL, aloe-emodin 16.4 μg/mL, rhein 45.4 μg/mL, emodin 41.8 μg/ mL, chrysophanol 123.9 μg/mL, physcion 14.5 μg/mL) was prepared in diluted to make six different concentrations including 1, 4/5, 3/5, 2/5, 1/5 and 1/10 of the original concentration as working solutions. All the standard solutions were stored in the refrigerator at 4°C before analysis. 

*Optimization of extraction procedure*

Eight samples from the same batch of WCA pill were weighted and extracted at three different temperature and five different solvents to obtain the optimum extraction procedure. The extraction time (30, 60 and 120 min) and solvents including the solution of methanol (50%, 100% v/v) and ethanol (20%, 60%, 100% v/v) were investigated. 

*Preparation of sample solutions*

One gram of pulverized powder was accurately weighed and ultrasonically extracted with 25 mL of methanol for 60 min in a conical flask, and then cooled to room temperature. The supernatant was filtered through a 0.22 μm syringe filter before analysis.

*Validation study*

Validation of this analytical method was performed in accordance with International Conference on Harmonization (ICH) guidelines. The method was validated in terms of linearity, limit of detection and quantification, precision and accuracy.

*Linearity, limit of detection (LOD) and limit of quanti*fi *cation (LOQ)*

The linearity study was achieved by diluting stock solution into a series of concentrations. The calibration curves were constructed for at least six concentrations in triplicate. The standard solutions were further diluted with methanol to provide a series of standard solutions with the appropriate concentrations. LOD and LOQ under the optimum chromatographic conditions were determined by injecting a series of standard solutions until the signal-to-noise (S/N) ratio for each compound was 3 for LOD and 10 for LOQ.

*Precision, accuracy, stability*

The precision of the method was determined for intra- and inter-day variations. The intra-day precision was performed by analyzing certain standard solutions for three times in a single day, while the inter-day precision was carried out in triplicate consecutive days. Three concentrations of standards were tested as follows: 160.80 μg/mL for naringin, 11.76 μg/mL for hesperidin, 161.68 μg/mL for neohesperidin, 173.28 μg/mL for magnolol, 140.16 μg/mL for honokiol, 27.16 μg/mL for dehydrodehydrocostus lactone, 28.74 μg/mL for costunolide, 3.29 μg/mL for aloe-emodin, 9.08 μg/mL for rhein, 8.36 μg/mL for emodin, 24.78 μg/mL for chrysophanol, and 2.92 μg/mL for physcion.

In order to evaluate the repeatability and stability of the detected components, according to the method of *Preparation of sample solutions *as above, six different samples prepared from the same batch of WCA pill were analyzed. The relative standard deviation (RSD) was taken as a measure of repeatability. Stability of sample solution was tested at room temperature. Stability of sample solution was analyzed at 0, 4, 8, 12, 24 and 48 h at room temperature, respectively.

Recovery tests were carried out to further investigate the accuracy of the method by adding three concentration levels (low, medium and high) of the mixed standard solutions into the known real sample. The resultant samples were then extracted and analyzed with the described method. The recovery of each component was calculated by the following formula:

Recovery (%) = (amount found - original amount) / amount added ×100%

Relative standard deviation was used to describe precision, repeatability, stability and recovery.

## Results and discussion

*Optimization of extraction procedure*

Various extraction methods, solvents and times were evaluated to obtain the best extraction efficiency. The results revealed that ultrasonic bath was better than refluxing the extraction considering more effective components and less interference. So the further experiments were carried out with ultrasonically extracting. Finally, the procedure of 60 min and 100% methanol was adopted as it produced much more peaks with higher response, little interference and better peak shapes.

*Optimization of chromatographic conditions*

**Figure 2:**
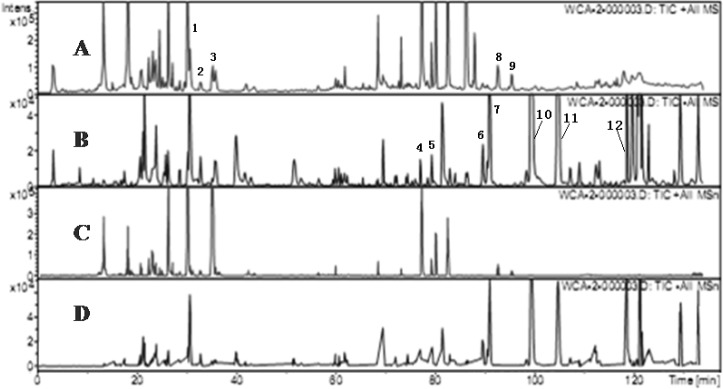
Chromatograms of WCA by HPLC-MS (A) TIC chromatogram in ESI positive mode.(B) TIC chromatogram in negative ESI mode. (C) TIC chromatogram of MS^n^ in ESI positive mode. (D) TIC chromatogram of MS^n^ in ESI positive mode.

To obtain chromatograms with good resolution of adjacent peaks, some HPLC analytical parameters including separation column, mobile phase and its elution mode were all investigated. Several trials were tried to achieve the good separation which included three kinds of C_18 _reversed-phase columns (Agilent ZOR-BAX, HiQ, Kromasil) and three gradient elution systems of methanol-water, acetonitrile-water and acetonitrile-methanol-water. The results indicated that a C_18_ Kromasil column (250 mm × 4.6 mm *i.d.*, 5 μm) and a C_18_ guard column (7.5 mm × 4.6 mm *i.d.*, 5 μm) were used. Meanwhile, a linear gradient elution of acetonitrile-methanol-water with 1% (v/v) acetic acid was selected since it permitted the best separation ability for all the samples investigated. The flow rate was 0.8 mL/min and the column temperature was maintained at 35°C. The DAD detector was employed at the wavelength range from 190 nm to 400 nm for obtaining a sufficient number of detectable peaks. The structures of twelve components were shown in Figure 2. Two hundred and thirty nm, 254 nm and 280 nm were selected by comparing all the chromatograms and the UV characteristic spectra of referenced compounds. Under the optimized conditions, all of the analytes were separated with good resolution.

*Identification of constituents in WCA extract*

**Figure 3:**
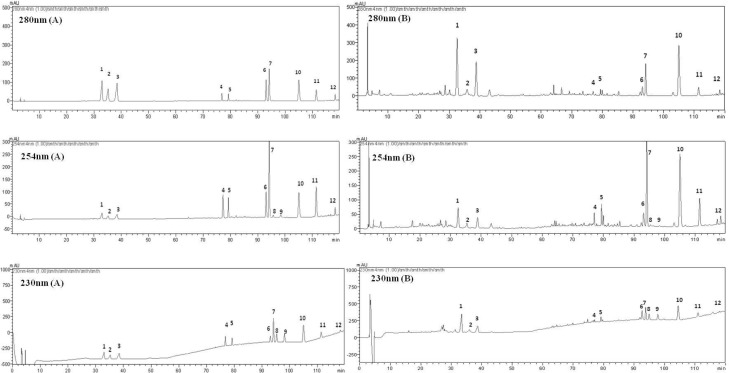
Typical chromatograms of the standard mixture (A) and WCA methanol extract (B) at 230, 254, 280 nm. (1) naringin; (2) hesperedin; (3) neohesperedin; (4) aloe-emodin; (5) rhein; (6) emodin; (7) honokiol; (8) dehydrodehydrocostus lactone; (9) costunolide; (10) magnolol; (11) chrysophanol; (12) physcion.

According to MS/MS data obtained by collision-induced dissociation, twelve components were unambiguously identified by the comparison of their retention times, MS data and UV spectra with the reference constituents. Figure 3 displayed the total ion chromatograms of WCA extract in positive and negative ion mode and the data of MS/MS of main components were summarized in Table 1. The detection of naringin, hesperidin, neohesperidin, costunolide and dehydrocostus lactone in positive mode were better than negative mode, while other seven components including five anthraquinones and two lignanoids were detected in negative mode (not in the positive condition). As for flavone glucosides, except the parent ion [M + H]^+^, protonated aglycones [M + H - 308]^+^ were the main fragment. The characters of m/z 581/273 presented the fragment of naringin, and m/z 611/303 was the character of hesperidin and neohesperidin. The fragmentation ions of naringin, hesperidin and neohesperidin were accordance with the data in other literatures (13, 14).

**Table 1:** The m/z values of ions of the reference compounds

**No.**	**Identification**	**Negative ion(m/z)**	**Positive ion(m/z)**
1	Naringin	-	581.1, 419.4, 273.2
2	Hesperidin	-	611.2, 449.5, 431.1
3	Neohesperidin	-	611.2, 449.5, 303.3
4	Aloe-emodin	269.1, 240.7	-
5	Rhein	283.2, 257.4, 239.3	-
6	Emodin	269.2, 241.4, 225.1	-
7	Honokiol	265.4, 224.3	-
8	Dehydrocostuslacton	-	233.2, 187.3
9	Costunolide	-	231.2, 185.6
10	Magnolol	265.2, 247.1	-
11	Chrysophanol	254.3, 225.7	-
12	Physcion	283.3, 268.4, 240.1	-

Costunolide and dehydrocostus lactone were sesquiterpene lactones in *R. Aucklandiae*. Except for the ion [M + H]^+^, m/z [M + H-46]^+^ were the main fragments ion in the detection. The appearance of [M + H-46]^+^ at m/z 187 and 185 were the fragment ion of sesquiterpene lactones, which the lactones ring opened and decarboxylated.

In the negative mode, magnolol and honokiol, a pair of isomers, both gave an [M-H]^-^ at m/z 265. In the ESI source, the fragments of lignanoids were observed in the side chain but not the parent nucleus. The fragment of magnolol was observed at m/z 247 which was the ion [M-H-H_2_O] ^-^, while the fragment of honokiol was observed at m/z 224 which was the ion [M-H-CH_2_CH=CH]^-^. The mass spectra of five anthraquinones were identified as aloe-emodin, rhein, emodin, chrysophanol and physcion. In the MS/MS spectrum, the fragment characters of the identified anthraquinones were accordance with the references, resulting from the loss of CO and CO_2_ (15).

*Validation of the chromatographic method Calibration curves, limits of detection and quantification*

The mixed standard stock solution containing twelve components was diluted to appropriate concentrations for plotting the calibration curves. Linearity of the method was investigated by analyzing six different concentration samples in triplicate. The calibration curves were achieved by plotting the peak areas versus the concentration of each analyte. The calculated results of linear calibration curve with R^2^ linear range were listed in Table 2. All the analytes showed good linearity (R^2^ > 0.999) in a relatively wide concentration range. The stock solutions of the analytes were further diluted with methanol to yield a series of appropriate concentrations for achieving LOD and LOQ. The results can be seen in Table 2.

**Table 2:** Statistics results of the method validation of the determination of twelve bioactive components in Weichang’an pill.

Compound	Linear range (μg mL^-1^**)**	LOD (μg mL^-1^)	LOQ (μg mL^-1^)	Precision			Repeatability (n = 6)			Stability (n = 3)		
				Intra-day (μg mL^-1^)	RSD (%)	Inter-day (μg mL^-1^)	RSD (%)	Mean (μg mL^-1^)	RSD (%)		Mean (μg mL^-1^)	RSD (%)	
Narirutin	80.40-804.0	0.24	0.80	157.97	0.68	156.88	2.05	186.92	1.70		184.03	1.57	
Hesperidin	5.88-58.8	0.26	0.88	11.88	0.91	11.92	0.98	8.80	2.29.		8.65	2.67	
Neohesperedin	80.84-808.4	0.19	0.65	157.77	0.74	158.82	0.67	86.42	0.97		85.67	1.61	
Aloe-emodin	1.64-16.4	0.049	0.16	3.33	1.33	3.24	0.83	2.15	2.14		2.15	1.98	
Rhein	4.54.-45.4	0.068	0.23	9.02	0.83	8.99	0.92	9.23	1.23		9.21	1.95	
Emodin	4.18-41.8	0.062	0.21	8.46	0.83	8.31	1.56	4.86	1.44		4.90	2.46	
Honokiol	70.08-700.8	0.35	1.17	137.71	0.82	141.25	1.26	81.57	0.87		81.78	1.13	
Dehydrodehydrocostus lactone	13.58-135.79	0.10	0.33	27.07	1.17	26.96	0.82	20.41	1.40		20.69	2.32	
Costunolide	14.37-143.70	0.057	0.19	28.19	1.21	28.35	1.28	16.38	1.58		16.51	2.61	
Magnolol	86.64-866.4	0.43	1.44	170.84	1.32	172.21	0.85	206.57	2.69		204.36	2.38	
Chrysophanol	12.39-123.90	0.13	0.44	5.76	0.78	5.83	0.92	18.54	1.50		18.57	2.03	
Physcion	1.46-14.55	0.045	0.15	2.97	0.80	2.94	1.99	6.00	1.24		6.02	1.76	

*Precision, accuracy and stability*

Table 2 showed the results of precision, repeatability and stability. The statistic data of the intra- and inter-day precision showed relative standard deviation (RSD) of twelve compounds less than 3%. For further evaluating the repeatability, six samples of WCA pill was analyzed under the selected conditions. The RSD values of peak area ranging from 1.13 to 2.67% showed that the sample solution was stable within 48 h at room temperature. Table 3 displayed the results of recovery test. For recovery test, mean recoveries of the standard substances were between 97.54% and 102.69%, with RSD less than 3% (n = 3). The results described above showed that the developed method was reliable for simultaneous determination of twelve bioactive components in WCA pill.

**Table 3:** The recovery data of twelve bioactive components in Weichang’an pill

**Compound**	**Initial amount (μg mL** ^-1^ **)**	**Added amount (μg mL** ^-1^ **)**	**Detected amount (μg mL** ^-1^ **)**	**Recovery (%)**
Narirutin	192.16	81.28	274.48	101.33
		179.08	370.16	99.41
		248.76	439.92	99.62
Hesperidin	8.12	4.08	12.12	98.02
		8.16	16.48	102.55
		12.24	20.56	101.61
Neohesperedin	82.84	44.84	125.88	98.47
		81.72	166.60	102.51
		124.48	209.56	101.86
Aloe-emodin	3.232	1.71	4.92	99.03
		3.42	6.64	99.85
		5.12	8.40	100.95
Rhein	11.96	6.16	18.28	102.69
		12.32	24.20	99.47
		18.48	30.20	98.71
Emodin	6.88	3.40	10.36	102.20
		6.80	13.84	102.44
		10.20	17.00	99.26
Honokiol	126.8	57.00	183.04	98.72
		116.76	244.04	100.43
		163.48	292.40	101.33
Dehydrodehydrocostus lactone	35.8	18.04	54.00	100.97
		36.08	71.64	99.38
		54.12	89.20	98.73
Costunolide	37.52	18.28	55.72	99.64
		36.56	73.40	98.16
		54.84	91.96	99.34
Magnolol	210.64	100.44	311.48	100.43
		217.08	430.32	101.27
		297.00	509.72	100.73
Chrysophanol	15.56	8.16	23.68	99.53
		16.32	32.28	102.53
		24.48	39.68	98.56
Physcion	4.56	2.44	6.92	96.71
		4.88	9.32	97.54
		7.32	11.76	98.42

*Sample analysis*

The proposed HPLC-DAD method was successfully applied to simultaneous determination of the twelve components in ten batches of WCA pill. All the contents were summarized in Table 4. Results showed that the contents of ten components including naringin, hesperidin, neohesperidin, magnolol, honokiol, aloe-emodin, rhein, emodin, chrysophanol and physcion have been obviously consistent in ten batch samples. However, there was a wide variation in the contents of costunolide and dehydrocostus lactone, two constituents from the principal herb *R. Aucklandiae*. In the Pharmacopoeia of China, the contents of magnolol and honokiol were used to be the basis for quality control of WCA pill. The analysis of the components from one composition herb cannot supply the sufficient evidence for the complicated system. Unlike the synthetic, traditional Chinese medicinal formula exerts the curative effects based on the synergic effects of the multi-components and multi-targets (16). Therefore, the quantitative analysis of more bioactive constituents from different composition herb in the formula was needed for the quality control of complex analytes. In this study, the quantitative analysis of the twelve components included the main bioactive constituents from the composition of the principal, the ministerial and the adjunctive and messenger herbs. This method improved the quality control level of WCA pill by simultaneous determination of the multiple active components in the products. In this formula, the contents of the main active components from the four herbs were above 1 mg/g and the total contents of naringin, hesperidin, neohesperidin from *F. Aurantii *and that of magnolol and honokiol from *C. Magnoliae officinalis *were higher than that of other constituents in WCA pill. Though *R. et R. Rhei *is not the major in the contents, it plays an important role in the formula treating IBS-D. Therefore, the quantity control of active components in *R. et R. Rhei *is the key to assess the overall efficacy. The principal herb *R. Aucklandiae *takes a large proportion in whole formula. Costunolide and dehydrocostus lactone, the volatile oil, account for a large proportion in *R. Aucklandiae *(17, 18)*. *Due to the original area of medicinal herb, the contents of these two ingredients varied from 1.1% to 2.1%. According to China pharmacopoeia, the total contents of costunolide and dehydrocostus lactone should not be less than 1.8%. Even though, *R. Aucklandiae *is principal drug in WCA, there had been no report about the contents of costunolide and dehydrocostus lactone in the products. The results of present study displayed that the difference existed in the contents of costunolide and dehydrocostus lactone in WCA samples of different batches. The contents of costunolide and dehydrocostus lactone ranged from 1.0 mg/g to 1.8 mg/g. There are two reasons which may lead to this inconsistency. On the one hand, it is because of the different original area of the herb, and on the other hand, it may be due to the loss of volatile components in the producing and reserving process. The multi-components quantitative analysis displayed an effective method to establish the standards for quality control of traditional Chinese medicine formula and to ensure the accuracy and efficiency in the manufacturing process of WCA pill.

**Table 4:** Quantitative determinations of twelve components in Weichang’an pill samples

**Components**	**Content (n = 3, mg g** ^-1^ **)**
**Narirutin**	4.32-5.02
**Hesperidin**	0.14-0.20
**Neohesperedin**	1.89-2.23
**Aloe-emodin**	0.078-0.082
**Rhein**	0.27-0.32
**Emodin**	0.16-0.18
**Honokiol**	2.88-3.25
**Dehydrodehydrocostus lactone**	0.52-0.97
**Costunolide**	0.47-0.88
**Magnolol**	5.00-5.38
**Chrysophanol**	0.37-0.42
**Physcion**	0.11-0.13

## Conclusion

The major active components identified in the extract of WCA pill including naringin, hesperidin, neohesperidin, magnolol, honokiol, costunolide, dehydrodehydrocostus lactone, aloe-emodin, rhein, emodin, chrysophanol and physcion are respectively from the principal herb *R. Aucklandiae*, the ministerial herbs *F. Aurantii *and *C. Magnoliae officinalis*, the adjunctive and messenger herb *R. et R. Rhei*. The analytical method developed in the present study is specific for the simultaneous quantification of twelve constituents in WCA pill. This readily available, rapid and reliable method is fit for the routine analysis of the complicated system and the precise quantity of the bioactive components in the formula lays the groundwork for the deep study on therapeutic basis and pharmacological function mechanism of traditional Chinese medicine formula.
